# Clinicopathologic characteristics of early gastric cancer according to specific intragastric location

**DOI:** 10.1186/s12876-019-0949-5

**Published:** 2019-02-08

**Authors:** Kyungeun Kim, Younghye Cho, Jin Hee Sohn, Dong-Hoon Kim, In Gu Do, Hyun Joo Lee, Sung-Im Do, Sangjeong Ahn, Hyoun Wook Lee, Seoung Wan Chae

**Affiliations:** 1 Department of Pathology, Kangbuk Samsung Hospital, Sungkyunkwan University School of Medicine, 29 Saemunan-ro, Jongno-gu, Seoul, 03181 South Korea; 2 U2 Hospital, Jangwon Medical Foundation, Seoul, South Korea; 3 Department of Pathology, Catholic Kwandong University College of Medicine, International St. Mary’s Hospital, Incheon, South Korea; 4 Department of Pathology, Samsung Changwon Hospital, Sungkyunkwan University School of Medicine, Changwon, Gyeongsangnam-do South Korea

**Keywords:** Stomach neoplasms, Neoplasm by site, Gastrectomy, Incidence

## Abstract

**Background:**

Although the incidence of early gastric cancer (EGC) continues to rise, there have been few studies on the intra-gastric distribution and locational characteristics of EGCs. In addition, there has been no attempt to visualize the intra-gastric distribution of EGCs using a merged tumor map.

**Methods:**

We investigated the anatomic distribution of 644 cases of EGCs and analyzed the correlation between clinicopathologic findings and location by dividing areas of the stomach vertically and transversely. Merged tumor maps were generated using 310 surgically resected cases.

**Results:**

Early gastric cancer was most commonly located in the antrum (57.5%) along the lesser curvature (37.8%). The intra-gastric distributions were similar in the merged tumor maps. Vertically, cancers of the middle third were associated with younger patient age, larger tumor size, and more frequent poorly differentiated (PD) or signet ring cell histology than cancers in other sites. Submucosal invasion was most frequently observed in the upper third. When divided transversely, tumors in the anterior or posterior wall showed more frequent PD or signet ring cell histology than those along the lesser or greater curvatures.

**Conclusions:**

EGC is the most prevalent in the antrum along the lesser curvature and has characteristic locational features, including histologic type, invasion depth, patient age, and tumor size. These results will improve the endoscopic detection rate of EGC and help to determine endoscopic resectability.

**Electronic supplementary material:**

The online version of this article (10.1186/s12876-019-0949-5) contains supplementary material, which is available to authorized users.

## Background

Gastric cancer is the fourth most common cancer and the second leading cause of cancer-related death worldwide [1]. In Korea, gastric cancer is the second most common cancer among the male population and the fourth most common cancer in the female population [2]. Early gastric carcinoma (EGC) is an invasive neoplasm confined to the gastric mucosa or submucosa irrespective of lymph node metastasis. EGC can be treated via endoscopic resection with an excellent survival rate and high quality of life for patients; thus, it is important to identify candidates for endoscopic resection [3, 4]. Endoscopic resectability is determined by various clinicopathologic factors, including tumor differentiation, tumor size, invasion depth, ulceration, and lymphovascular invasion, which vary depending on the intragastric location of EGCs [5]. However, studies of the intra-gastric distribution of EGCs or their locational characteristics have been rare [6, 7]. In addition, there have been no attempts to visualize the real distribution of EGCs using merged tumor maps.

The stomach epithelium originates from the foregut endoderm and becomes regionalized along the proximal-distal axis during development to give rise to distinct functional regions or chambers [8]. Anatomically, the stomach is classified into the cardia, fundus, body, antrum, and pylorus, as well as the lesser and greater curvatures. Most prior studies on the locational characteristics of gastric cancers have divided the stomach into more general categories of proximal versus distal or cardiac versus non-cardiac [6, 9–12]. As the incidence of proximal gastric cancer grows globally, many studies have focused on the evaluation of characteristics of proximal gastric cancer. These studies have revealed that while proximal gastric cancer is typically more aggressive and has a poor prognosis, the most prevalent gastric cancer location is the antrum [6, 9–15]. Therefore, further knowledge regarding the clinicopathologic features of gastric cancer according to detailed intragastric location is needed to aid in selecting appropriate treatment strategies.

We investigated the anatomic distribution of EGC by generating a merged tumor map and then analyzed the correlation between clinicopathologic findings and location based on detailed segmentation of the stomach.

## Method

### Patients and clinical data

This retrospective study was approved by the Institutional Review Board of Kangbuk Samsung Hospital. A total of 310 gastrectomy specimens and 334 endoscopic submucosal dissection (ESD) specimens of EGC resected at Kangbuk Samsung Hospital between January 2011 and December 2014 was included. Subtotal and total gastrectomy were performed in 264 (85.2%) and 45 (14.5%) patients, respectively. One patient (0.3%) underwent a proximal gastrectomy. Clinical data including age, sex, and follow-up findings were obtained from electronic medical records.

### Gross examination of gastrectomy specimens and generation of merged tumor map

**Fig. 1:**
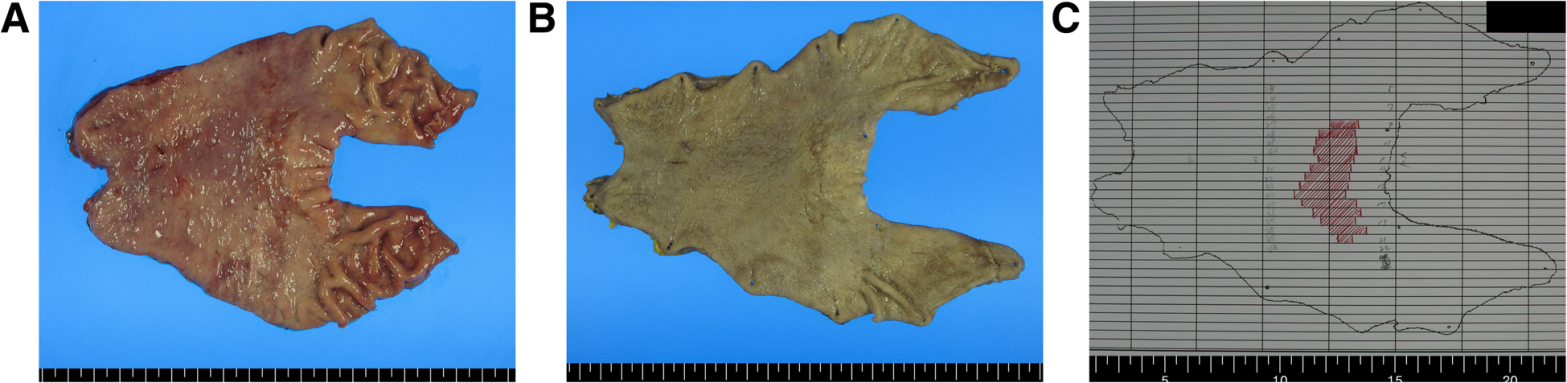
Gross fresh (**a**) and formalin-fixed (**b**) subtotal gastrectomy specimens and tumor map generated after microscopic examination (**c**). The stomach is opened along the greater curvature, and the proximal part is on the right (**a**). An ill-defined slightly depressed lesion is seen in the low body along the lesser curvature (**b**). The tumor is marked by red in the tumor map after microscopic examination (**c**)

Fresh gastrectomy specimens were photographed after opening along the greater or lesser curvature (Fig. 1a). After fixation in 10% buffered formalin solution overnight, fixed gastrectomy specimens were photographed, and the definite tumor location and extent was determined (Fig. 1b and c). Gross characteristics including tumor location, tumor size, and EGC type were recorded. Vertical locations were recorded as gastroesophageal junction, cardia, fundus, high body, mid body, low body, antrum, and pylorus. To analyze clinicopathologic parameters, three groups were used: upper third, from the gastroesophageal junction to the high body; middle third, mid body and low body; lower third, antrum and pylorus. Transverse locations were recorded as the lesser curvature, greater curvature, anterior wall, and posterior wall. The transverse location could not be determined in cases located in the cardia, fundus, or gastroesophageal junction due to circular tumor shape.

Images of tumor maps were merged using the ImageJ program (http://imagej.net/) according to type of operation and opening method. Among 310 cases, 31 cases opened in unusual ways were excluded from the merged tumor map. Finally, 250 cases opened along the greater curvature and 29 cases opened along the lesser curvature were included. To adjust the size and position of images, the bUnwarpJ plugin of ImageJ was used. After registration, the tumor area was manually labeled using the rectangular selection tool in ImageJ. The labeled images were saved individually. Then, merged stack images were created using ImageJ’s Z Project menu.

### Gross examination of endoscopic submucosal dissection specimens

ESD specimens were photographed after fixation in 10% buffered formalin solution overnight. After applying dye to the deep and lateral resection margins, the specimens were cut at 2-mm increments and mounted on glass slides. Tumor locations were determined based on endoscopic findings. Gross characteristics including tumor location and tumor size were recorded in the same manner as for gastrectomy specimens.

### Microscopic evaluation

All gastrectomy and ESD cases were microscopically reviewed to determine the histological classification, tumor differentiation, depth of invasion, presence of lymphovascular invasion, presence of perineural invasion, and presence of adenomatous background [13]. In cases with two or more EGC lesions in the same specimen, tumor characteristics (including histologic type and depth of invasion) of the EGC lesion with the deepest tumor invasion were used. Tumor stage was assigned according to the 2010 AJCC Tumor Node Metastasis staging system [1].

### Statistical analysis

Data were analyzed using PASW Statistics 18 (SPSS Inc., Chicago, IL, USA) software. Crosstabs, Pearson’s chi-square test, and Fisher’s exact test were used as needed. Differences were regarded as statistically significant if *P* < 0.05.

## Results

**Fig. 2:**
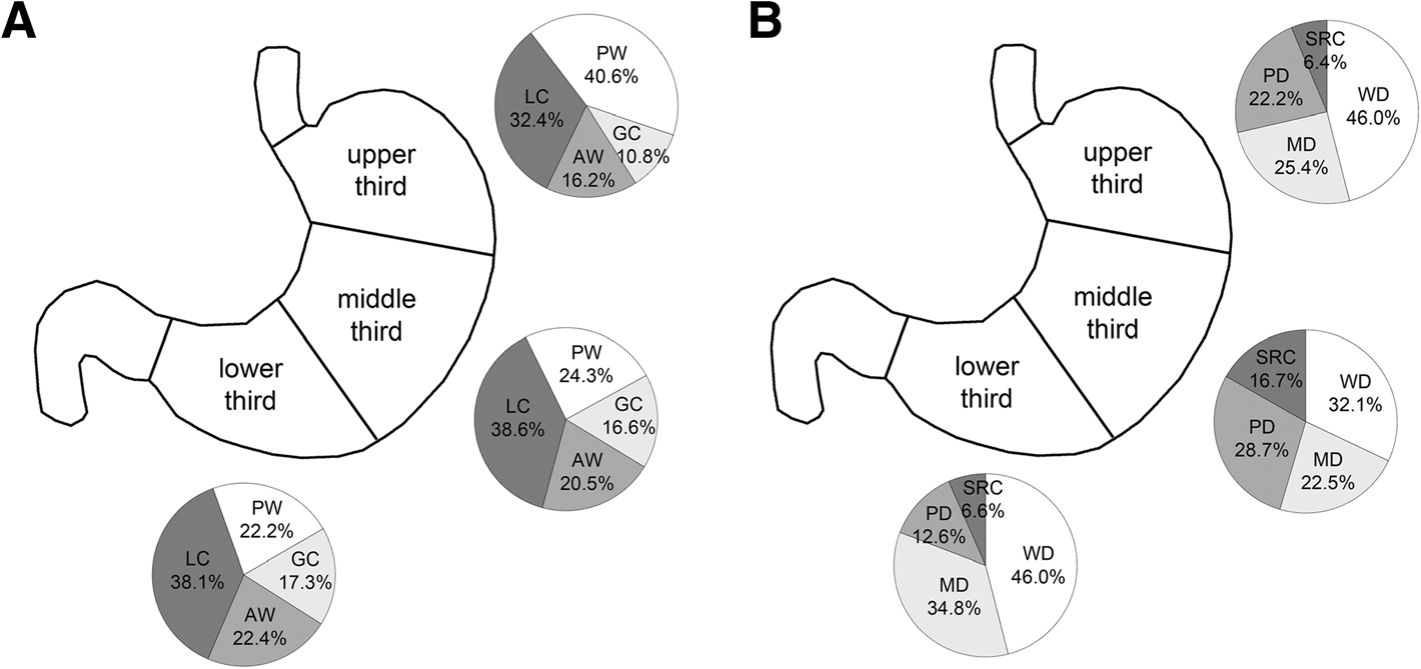
Transverse location (**a**) and histologic distribution (**b**) of early gastric carcinoma according to vertical location. The transverse location is indicated as posterior wall (PW), greater curvature (GC), anterior wall (AW), and lesser curvature (LC) (**a**). The middle third shows more frequent poorly differentiated (PD) adenocarcinoma or signet ring cell carcinoma (SRC) histology compared to upper or lower thirds, which has predominent well diffrentiated (WD) or moderatedly differentiated (MD) histology (**b**)

**Fig. 3:**
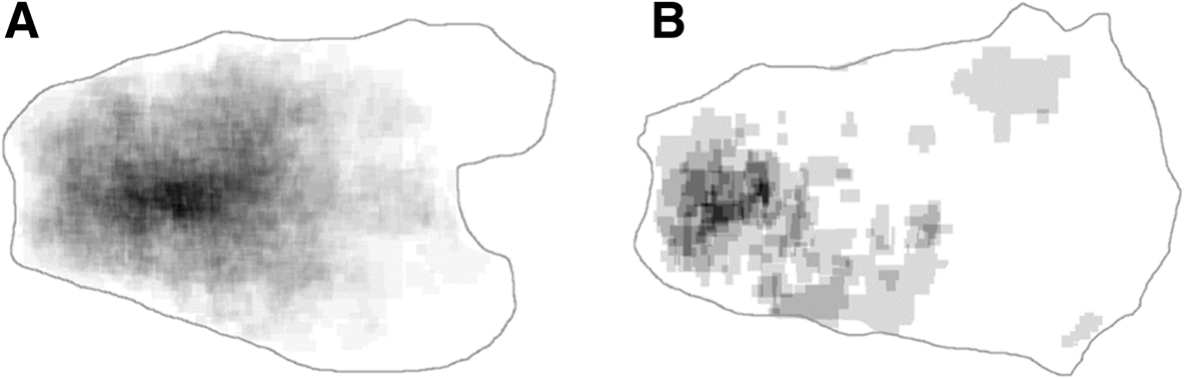
Merged tumor maps generated according to surgical opening along the greater (**a**) or lesser (**b**) curvatures. Tumors are most often concentrated in the antrum and low body along the lesser curvature (**a**). Along the greater curvature, tumors are located only in the antrum and low body, with preservation of the mid and high body (**b**)

The clinicopathologic data for all 644 patients with EGC are given in Table 1. The median age was 61 (range: 29 to 87 years) and 67 years (range: 30 to 89 years) in patients who underwent gastrectomy and ESD, respectively. The mean lesion size was 3.17 cm (range: 0.2 to 14.5 cm) and 1.89 cm (range: 0.1 to 7.8 cm) in patients who underwent gastrectomy and ESD, respectively. More than half of cases had tumors located in the antrum (57.5%) (Table 2). The most common EGC location was the antrum along the lesser curvature (21.9%), followed by the anterior wall of the antrum (12.9%) and the posterior wall of the antrum (12.7%). When dividing cases into three groups according to vertical location, tumors in the upper third were most commonly located in the posterior wall, while tumors in the middle and lower third showed lesser curvature predominance (Fig. 2a). The merged tumor map with gastrectomy cases showed a hot spot in the antrum and low body along the lesser curvature (Fig. 3a). The tumor map with cases opened along the lesser curvature also revealed a hot spot in the antrum along the greater curvature with a more distal location (Fig. 3b).

**Table 1:** Clinicopathologic features of 644 patients with early gastric cancer

Variables	Gastrectomy (*n* = 310)	Endoscopic resection (*n* = 334)	Total (*n* = 644)
Sex			
Male	211 (68.1)	256 (76.6)	467 (72.5)
Female	99 (31.9)	78 (23.4)	177 (27.5)
Size (cm)			
≤ 2	103 (33.2)	217 (65.0)	320 (49.7)
2.1–3.0	78 (25.2)	71 (21.2)	149 (23.1)
> 3	129 (41.6)	46 (13.8)	175 (27.2)
Gross type			
Elevated	56 (18.1)	89 (26.7)	145 (22.5)
Flat	121 (39.0)	133 (39.8)	254 (39.4)
Depressed	133 (42.9)	112 (33.5)	245 (38.1)
pT stage			
1a	156 (50.3)	269 (80.5)	425 (66.0)
1b	154 (49.7)	65 (19.5)	219 (34.0)
Histologic type			
Well differentiated	58 (18.7)	206 (61.7)	264 (41.0)
Moderately differentiated	91 (29.4)	99 (29.6)	190 (29.5)
Poorly differentiated	99 (31.9)	21 (6.3)	120 (18.6)
Signet ring cell carcinoma	58 (18.7)	5 (1.5)	63 (9.8)
Mucinous adenocarcinoma	3 (1.0)	1 (0.3)	4 (0.6)
Lymphoepithelioma-like	1 (0.3)	2 (0.6)	3 (0.5)
Lymphatic invasion	46 (14.8)	23 (6.9)	69 (10.7)
Vascular invasion	5 (1.6)	2 (0.6)	7 (1.1)
Perineural invasion	6 (1.9)	0 (0)	6 (0.9)
Adenomatous background	15 (4.8)	80 (24.0)	95 (14.8)

Data are presented as number (%)

**Table 2:** Location of 644 cases of early gastric cancer

	Greater curvature	Lesser curvature	Anterior wall	Posterior wall	Total
GEJ, cardia, fundus^a^					27 (4.2)^a^
High body	4 (0.6)	12 (1.9)	6 (0.9)	15 (2.3)	37 (5.7)
Mid-body	8 (1.2)	29 (4.5)	8 (1.2)	17 (2.6)	62 (9.6)
Low body	27 (4.2)	52 (8.1)	35 (5.4)	34 (5.3)	148 (23.0)
Antrum	64 (9.9)	141 (21.9)	83 (12.9)	82 (12.7)	370 (57.5)
Total cases	103 (16.7)	234 (37.8)	132 (21.4)	148 (24.0)	

Data are presented as number (%)

*ESD* endoscopic submucosal dissection, *GEJ* gastroesophageal junction

^a^The transverse location cannot be determined

**Fig. 4:**
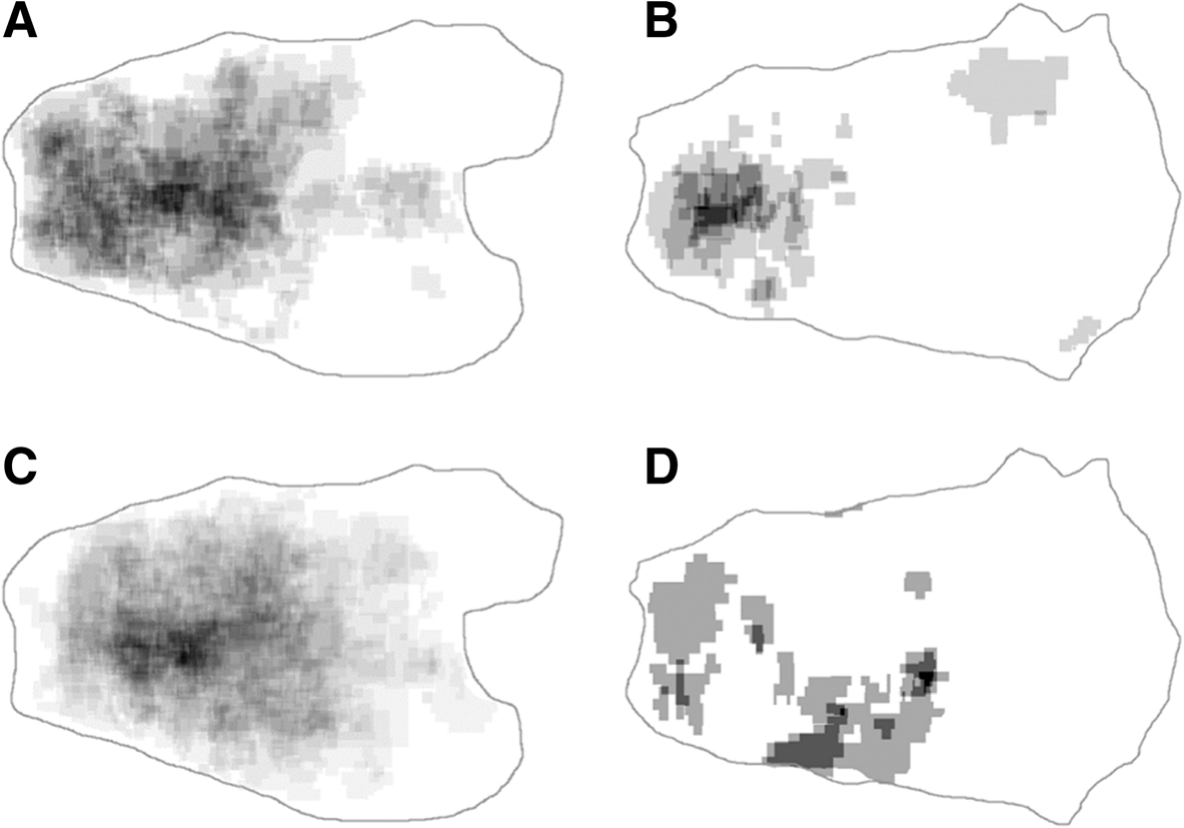
Tumor distribution by tumor differentiation. Tumor maps are merged according to surgical opening along greater (**a**, **c**) or lesser (**b**, **d**) curvatures. The highest concentrations of well and moderately differentiated adenocarcinoma (**a**, **b**) are located in a lower part than most poorly differentiated adenocarcinomas and signet ring cell carcinomas (**c**, **d**)

When dividing the cases into three vertical groups according to tumor epicenter, there were differences between groups in tumor size, age, invasion depth, and histologic differentiation (Table 3). The middle third showed poorly differentiated (PD) adenocarcinoma or signet ring cell carcinoma (SRC) most frequently, while in the remaining two thirds well-differentiated (WD) adenocarcinoma was the most common histologic pattern (Table 3, Additional file 1: Table S1 and Fig. 2b). Similar distributional differences were also observed in the merged tumor maps. PD adenocarcinoma and SRC were more concentrated in the middle third (Fig. 4c and d) compared to WD and moderately differentiated (MD) tumors (Fig. 4a and b). In addition, the middle third exhibited the largest mean tumor size and the youngest mean age. Submucosal invasion was most frequently observed in the upper third (Table 3). Gender, gross type, lymphovascular invasion, perineural invasion, adenomatous background, and lymph node metastasis were not significantly different according to vertical location.

**Table 3:** Clinicopathological features according to the vertical location of early gastric cancer

Variables	Upper third (*n* = 64, 9.9%)	Middle third (*n* = 210, 32.6%)	Lower third (*n* = 370, 57.5%)	*P*-value
Size (cm)	2.77 ± 2.09	2.96 ± 2.06	2.21 ± 1.54	< 0.001
Age (years)	63.56 ± 12.46	60.28 ± 11.70	64.24 ± 11.11	< 0.001
Sex				0.249
Male	52 (81.3)	149 (71.0)	266 (71.9)	
Female	12 (18.8)	61 (29.0)	104 (28.1)	
pT stage				0.001
1a	30 (46.9)	134 (63.8)	261 (70.5)	
1b	34 (53.1)	76 (36.2)	109 (29.5)	
Gross type				0.263
Elevated	18 (28.1)	40 (19.0)	87 (23.5)	
Flat	27 (42.2)	91 (43.3)	136 (36.8)	
Depressed	19 (29.7)	79 (37.6)	147 (39.7)	
Histologic type^a^				< 0.001
WD	29 (46.0)	67 (32.1)	168 (46.0)	
MD	16 (25.4)	47 (22.5)	127 (34.8)	
PD / Signet ring cell carcinoma	18 (28.6)	95 (45.4)	70 (19.2)	
Lymphatic invasion	6 (9.4)	21 (10.0)	42 (11.4)	0.823
Venous invasion	0 (0)	3 (1.4)	4 (1.1)	0.628
Perineural invasion	1 (1.6)	1 (0.5)	4 (1.1)	0.658
Adenomatous background	12 (18.8)	30 (14.3)	53 (14.3)	0.636
Lymph node metastasis^b^	2 (5.4)	12 (9.3)	20 (12.2)	0.420

Data are presented as number (%) or mean ± standard deviation

*SD* standard deviation, *WD* well differentiated, *MD* moderately differentiated, *PD* poorly differentiated

^a^Mucinous adenocarcinoma and carcinoma with lymphoid stroma were excluded from this analysis

^b^Lymph node metastasis was analyzed in only 310 gastrectomy specimens

When dividing the stomach transversely, the lesser curvature (37.8%) was the most common site for EGCs, and the greater curvature (16.7%) was the least (Table 2) common site. Tumors located in the anterior or posterior wall showed a higher frequency of PD adenocarcinoma/SRC histology than those located along the greater or lesser curvatures (Table 4). These histologic differences were not demonstrated on the merged tumor map because the frequency of tumors located along the lesser curvature was remarkably high (Fig. 4). Tumors located along the lesser curvature showed the largest tumor size and most commonly had an adenomatous background. Tumors located in the posterior wall were associated with the youngest mean age (Table 4). The remaining clinicopathologic features were not significantly different according to transverse location.

**Table 4:** Clinicopathological features according to the transverse location of early gastric cancer

Variables	Greater curvature	Lesser curvature	Anterior wall	Posterior wall	*P*-value
Size (cm)	2.02 ± 1.23	2.81 ± 2.13	2.55 ± 1.79	2.28 ± 1.45	0.001
Age (years)	64.72 ± 11.56	63.26 ± 11.17	62.74 ± 0.89	60.59 ± 12.65	0.036
Sex					0.343
Male	79 (76.7)	172 (73.5)	88 (66.7)	105 (70.9)	
Female	24 (23.3)	62 (26.5)	44 (33.3)	43 (29.1)	
pT stage					0.171
1a	66 (64.1)	169 (72.2)	82 (62.1)	96 (64.9)	
1b	37 (35.9)	65 (27.8)	50 (37.9)	52 (35.1)	
Gross type					0.275
Elevated	31 (30.1)	46 (19.7)	28 (21.2)	30 (20.3)	
Flat	40 (38.8)	88 (37.6)	57 (43.2)	61 (41.2)	
Depressed	32 (31.1)	100 (42.7)	47 (35.6)	57 (38.5)	
Histologic type^a^					0.041
WD	49 (48.5)	111 (47.6)	43 (32.8)	49 (33.6)	
MD	36 (35.6)	61 (26.2)	42 (32.1)	45 (30.8)	
PD / Signet ring cell carcinoma	16 (15.9)	61 (26.2)	46 (35.1)	52 (35.6)	
Lymphatic invasion	14 (13.6)	20 (8.5)	15 (11.4)	15 (10.1)	0.545
Venous invasion	1 (1.0)	1 (0.4)	3 (2.3)	2 (1.4)	0.448
Perineural invasion	0 (0)	2 (0.9)	2 (1.5)	1 (0.7)	0.638
Adenomatous background	13 (12.6)	46 (19.7)	15 (11.4)	16 (10.8)	0.047
Lymph node metastasis^b^	6 (15.0)	6 (5.6)	9 (11.1)	12 (14.0)	0.201

Data are presented as number (%) or mean ± standard deviation

*SD* standard deviation, *WD* well differentiated, *MD* moderately differentiated, *PD* poorly differentiated

^a^Mucinous adenocarcinoma and carcinoma with lymphoid stroma were excluded from this analysis

^b^Lymph node metastasis was analyzed in only 310 gastrectomy specimens

## Discussion

This is the first study in Korea to provide detailed distribution and locational characteristics of all EGCs, including both surgically and endoscopically resected EGCs. We confirmed that the antrum (57.5%) and lesser curvature (37.8%) were the most common sites of longitudinally and transversely classified EGCs, respectively. These results are similar to those of a previous Korean study of gastric cancers which revealed that gastric cancer is most commonly located in the lower third (56.0%) of the stomach [15]. In a study performed in the USA, 32.2% of all EGCs were located in the antrum. In comparison, advanced and early gastric cancers in Koreans were more concentrated in the antrum [14]. We also found that EGCs with PD or SRC histology more commonly occurred in the middle third vertically and in the anterior or posterior wall transversely than in other locations. Submucosal invasion was most frequent in the upper third. Our results may serve as a good reference for evaluating endoscopic resectability as well as increasing the endoscopic detection rate of EGC. This is also the first attempt to visualize EGC frequency by creating merged tumor maps reflecting the actual location and extent of EGCs. Additional multi-center experiments with more tumor maps, including endoscopically resected cases, will be helpful in creating a Korean EGC map.

Studies on the locational characteristics of EGC are rare, although there have been several previous reports of locational characteristics of all gastric cancers [6, 7, 16–21]. A Chinese study, the only study including both surgically and endoscopically resected EGCs, divided the stomach into proximal and distal portions and revealed that proximal gastric cancer had smaller tumor size, deeper invasion depth, less frequent lymph node metastasis, and less frequent poorly cohesive histology than distal gastric cancers [6]. Our findings were consistent in that the upper third showed the deepest invasion, but the remaining features were not comparable. It is unclear whether the distal classification matched the middle and lower third categories of our study. A previous Korean study reported a similar result that tumors in the mid-to-upper portion of the stomach were larger and exhibited more frequent submucosal invasion than those in the lower portion, but this study included only endoscopically resected EGCs [7]. Our results present more detailed locational characteristics and include EGC cases regardless of treatment modality. These are strengths that distinguish our study from the previous studies mentioned above.

Our results showed that EGCs in the middle third longitudinally and in the anterior or posterior wall transversely were more likely to represent PD adenocarcinoma or SRC than tumors found at other sites. In a previous study comparing the locational characteristics of gastric cancer from two cohorts in Koreans and Americans, the Korean cohort more frequently had undifferentiated cancers in the upper and middle thirds than in the lower third. However, there was no significant histologic difference between longitudinal locations in the American cohort [19]. A Chinese study also reported that PD type was found more frequently in the middle third than in the upper and lower thirds [22]. Based on our results and the abovementioned previous reports, gastric cancers in the middle and upper thirds have different histologic types than those of the lower third in Asian people including Koreans. These results suggest that gastric cancers occur via different carcinogenesis pathways depending on the intra-gastric location. Further studies investigating precancerous lesions according to detailed intra-gastric location are necessary, including not only the atrophic gastritis-intestinal metaplasia-dysplasia sequence but also signet ring cell carcinoma in situ lesions.

Gastric cancers located in the upper third, especially the cardia and gastroesophageal junction, are known to have a poor prognosis independent of stage [16]. In our results, submucosal invasion was most frequently found in the upper third. Akashi et al. reported that the cardia had looser smooth muscle bundles and more frequent large lymphatics in the muscularis mucosae layer than other gastric sites, which is presumed to be one reason for more frequent submucosal invasion in the cardia [23]. Endoscopy in the mid-to-upper stomach is technically difficult, which may also result in decreased early detection [7]. However, our study and also previous reports included a small number of upper third cancers, which limits investigation of the frequent submucosal invasion seen in this area.

This study has a few limitations. First, since only tumor maps of surgically resected cases were used in the merging processes of tumor maps, the merged tumor maps in this study reflected only the locations of surgically resected EGC cases rather than entire EGCs. Second, this study included no information about lymph node metastasis status after ESD procedures, tumor recurrence status, or patient survival.

## Conclusion

In conclusion, EGC was most commonly located in the antrum along the lesser curvature in Koreans. Younger patient age, larger tumor size, and more frequent PD adenocarcinoma and SRC were observed in the middle third on vertical location analysis. Submucosal invasion was most frequently observed in the upper third. Regarding transverse locations, anterior and posterior wall tumors showed more frequent PD adenocarcinoma and SRC. These location-specific features may be helpful in determining treatment options for patients with EGC.

## Additional file

Additional file 1: **Table S1.** Transverse location and histologic differentiation according to vertical location. (DOCX 15 kb)

## Abbreviations

EGC: Early gastric carcinoma
ESD: Endoscopic submucosal dissection
MD: Moderately differentiated
PD: Poorly differentiated
PTD: Proximal, transitional, and distal
SRC: Signet ring cell carcinoma
WD: Well differentiated

## References

[1] Edge SB, Byrd DR, Compton CC, April GF, Greene FL, Trotti A (2010). AJCC cancer staging manual.

[2] Jung KW, Won YJ, Kong HJ, Lee ES (2018). Prediction of Cancer incidence and mortality in Korea, 2018. Cancer Res Treat.

[3] Suzuki H, Oda I, Abe S, Sekiguchi M, Mori G, Nonaka S, Yoshinaga S, Saito Y (2016). High rate of 5-year survival among patients with early gastric cancer undergoing curative endoscopic submucosal dissection. Gastric Cancer.

[4] Yamashita K, Sakuramoto S, Shibata T, Nemoto M, Mieno H, Katada N, Kikuchi S, Watanabe M (2013). Survival outcome of laparoscopic gastrectomy for clinical early (cT1) gastric cancer. Surg Today.

[5] Japanese Gastric Cancer A (2011). Japanese gastric cancer treatment guidelines 2010 (ver. 3). Gastric Cancer.

[6] Huang Q, Fang C, Shi J, Sun Q, Wu H, Gold JS, Weber HC, Guan W, Zhang Y, Yu C (2015). Differences in Clinicopathology of early Gastric carcinoma between proximal and distal location in 438 Chinese patients. Sci Rep.

[7] Kang DH, Choi CW, Kim HW, Park SB, Kim SJ, Nam HS, Ryu DG (2017). Location characteristics of early gastric cancer treated with endoscopic submucosal dissection. Surg Endosc.

[8] Kim TH, Shivdasani RA (2016). Stomach development, stem cells and disease. Development.

[9] Kim DY, Joo JK, Ryu SY, Park YK, Kim YJ, Kim SK (2004). Clinicopathological characteristics of patients with proximal third gastric carcinoma. Acta Chir Belg.

[10] Park JC, Lee YC, Kim JH, Kim YJ, Lee SK, Shin SK, Hyung WJ, Noh SH, Kim CB (2010). Clinicopathological features and prognostic factors of proximal gastric carcinoma in a population with high helicobacter pylori prevalence: a single-center, large-volume study in Korea. Ann Surg Oncol.

[11] Amini N, Spolverato G, Kim Y, Squires MH, Poultsides GA, Fields R, Schmidt C, Weber SM, Votanopoulos K, Maithel SK (2015). Clinicopathological features and prognosis of gastric cardia adenocarcinoma: a multi-institutional US study. J Surg Oncol.

[12] Piso P, Werner U, Lang H, Mirena P, Klempnauer J (2000). Proximal versus distal gastric carcinoma--what are the differences?. Ann Surg Oncol.

[13] Bosman FT, Carneiro F, Hruban RH, Theise ND (2010). WHO classification of Tumours of the digestive system.

[14] Crane SJ, Richard Locke G, Harmsen WS, Diehl NN, Zinsmeister AR, Joseph Melton L, Romero Y, Talley NJ (2007). The changing incidence of oesophageal and gastric adenocarcinoma by anatomic sub-site. Aliment Pharmacol Ther.

[15] Jeong O, Park YK (2011). Clinicopathological features and surgical treatment of gastric cancer in South Korea: the results of 2009 nationwide survey on surgically treated gastric cancer patients. J Gastric Cancer.

[16] Petrelli F, Ghidini M, Barni S, Steccanella F, Sgroi G, Passalacqua R, Tomasello G (2017). Prognostic role of primary tumor location in non-metastatic Gastric Cancer: a systematic review and meta-analysis of 50 studies. Ann Surg Oncol.

[17] Marano L, Petrillo M, Pezzella M, Patriti A, Braccio B, Esposito G, Grassia M, Romano A, Torelli F, De Luca R, et al. Applicability of the proposed Japanese model for the classification of Gastric Cancer location: the "PROTRADIST" retrospective study. J Invest Surg. 2017;30(3):210–16. 10.1080/08941939.2016.1230248 27690693

[18] Kinami S, Fujimura T, Ojima E, Fushida S, Ojima T, Funaki H, Fujita H, Takamura H, Ninomiya I, Nishimura G (2008). PTD classification: proposal for a new classification of gastric cancer location based on physiological lymphatic flow. Int J Clin Oncol.

[19] Shim JH, Song KY, Jeon HM, Park CH, Jacks LM, Gonen M, Shah MA, Brennan MF, Coit DG, Strong VE (2014). Is gastric cancer different in Korea and the United States? Impact of tumor location on prognosis. Ann Surg Oncol.

[20] Adachi Y, Shiraishi N, Inomata M, Yasuda K, Hirabayashi Y, Kitano S (2000). Location of tumor and distribution of lymph node metastasis in gastric cancer: lesser curve or greater curve. Int J Surg Investig.

[21] Shida A, Fujioka S, Kawamura M, Takahashi N, Ishibashi Y, Nakada K, Mitsumori N, Omura N, Yanaga K (2014). Prediction of lymph node metastasis in patients with submucosa-invading early gastric cancer. Anticancer Res.

[22] Yu J, Zhao Q (2009). The demographic characteristics of histological types of gastric cancer with gender, age, and tumor location. J Gastrointest Cancer.

[23] Akashi Y, Noguchi T, Nagai K, Kawahara K, Shimada T (2011). Cytoarchitecture of the lamina muscularis mucosae and distribution of the lymphatic vessels in the human stomach. Med Mol Morphol.

