# Supplementary material for: Clinicopathologic characteristics of early gastric cancer according to specific intragastric location
Source: BMC Gastroenterol. 2019 Feb 8;19:24. doi: 10.1186/s12876-019-0949-5 (PMC6368692; doi:10.1186/s12876-019-0949-5)
Supplement: Supplementary file 1 — Table S1. Transverse location and histologic differentiation according to vertical location. (DOCX 15 kb) [file 12876_2019_949_MOESM1_ESM.docx]

| **Table S1.** Transverse location and histologic differentiation according to vertical location | | | | |
| --- | --- | --- | --- | --- |
| Variables | Upper third | Middle third | Lower third | *p*-value |
| Transverse location |  |  |  | 0.044 |
| posterior wall | 15 (40.6) | 51 (24.3) | 82 (22.2) |  |
| anterior wall/ LC/GC | 22 (59.4) | 159 (75.7) | 288 (77.8) |  |
| Histologic type^*^ |  |  |  | <0.001 |
| PD / signet ring cell carcinoma | 18 (28.6) | 95 (45.4) | 70 (19.2) |  |
| WD/MD | 45 (71.4) | 114 (54.6) | 295 (80.8) |  |
| LC = lesser curvature; GC = greater curvature; PD = poorly differentiated; WD = well differentiated; MD = moderately differentiated.  ^*^Mucinous adenocarcinoma and carcinoma with lymphoid stroma were excluded from this analysis. | | | | |
